# Supplementary material for: A quantum Hopfield associative memory implemented on an actual quantum processor
Source: Sci Rep. 2021 Dec 3;11:23391. doi: 10.1038/s41598-021-02866-z (PMC8642452; doi:10.1038/s41598-021-02866-z)
Supplement: Supplementary file 1 — Supplementary Information. [file 41598_2021_2866_MOESM1_ESM.pdf]

Supplementary Information to: A Quantum Hopfield Associative Memory Implemented on an Actual Quantum Processor

Nathan Eli Miller[1,*] **and Saibal Mukhopadhyay**[1]

[1]Georgia Institute of Technology, School of Electrical and Computer Engineering, Atlanta, GA, 30332, USA
[*]nathan.miller@gatech.edu

Gate Complexity of the Quantum Neuron Designs

One of the principal advantages of our proposed quantum neuron design as opposed to the quantum neuron design by Cao et al.[1] is its improvement in the gate complexity of the neuron circuit. To quantify gate complexity, we consider the basis gates $\{CNOT, ID, R_z, SX, X\}$, which are the basis gates for IBMQ hardware.[2] In the quantum neuron circuits in Figs. 1 and 2, we see the gates CR_y, R_y, CY, R_z, and $SWAP$ which must be decomposed to the basis gates to be executed in IBMQ hardware. The decomposition of these gates corresponds to 10, 4, 3, 1 and 3 basis gates respectively.[3] For example, the CY gate decomposes to the basis gate sequence $R_z(-\frac{\pi}{2}) \rightarrow CNOT \rightarrow R_z(\frac{\pi}{2})$ where the R_z gates are performed on the target qubit of the CY gate and the control and target of the $CNOT$ gate are the same as the original CY gate. Note there are some instances where these numbers reduce, such as $CR_y(\pi)$ only requiring 8 gates to perform due to simplifications permitted by the rotation angle π as opposed to a more complex angle, but in general we will assume that these edge cases occur infrequently.

The Cao et al.[1] neuron circuit shown in Fig. 1 requires $2(n-1)$ CR_y rotations, two R_y rotations for the β terms, one CY, and one R_z per update. If the update fails to produce the correct rotation on the output qubit, an additional R_y rotation is used to reverse the rotation, and the primary construction is repeated f additional times until a successful rotation is achieved. Once success is achieved, a $SWAP$ gate is used to overwrite the original qubit, and the ancilla qubit is reset if possible. This process occurs for each of the u updates the system performs. Therefore, the gate complexity of this circuit in the IBMQ hardware basis gates is equal to $[20n(f+1)-4f-5]u$ or approximately $O(nuf)$, not including non-unitary operations such as measurement and reset. It is also worth noting the gate complexity in terms of $CNOT$ gates and single-qubit gates, as $CNOT$ gates are the more resource intensive of the standard basis gates. The RUS neuron requires $[16n(f+1)-f-5]u$ single qubit gates and $[4n(f+1)-3f]u$ $CNOT$ gates.

The gate complexity of the Cao et al.[1] neuron also increases significantly if the circuit is recursively applied in order to create a rotation function with a sharper slope described by $R_y(2(arctan(tan^2\phi))^{\circ k})$ where k describes the number of recursions. We benchmark our system against the $k=1$ system in this study, but it is also useful to compare the qubit and time overhead of the full RUS system. Increasing k helps to decrease the error rate ε and increase the success rate $1-\nu$ of the RUS system. To simulate an associative memory of n neurons with t updates up to error ε and success probability at least $1-\nu$, with weights and biases discretized with resolution δ, Cao et al. cite their expected runtime as $O(\frac{n^{2.075}t}{\delta^{2.075}\varepsilon^{3.15}}log(\frac{t}{\nu}))$ and their qubit overhead as $O(n+log\frac{n}{\delta\varepsilon^{1.75}})$.[1]

For our proposed quantum neuron design, we cut the number of required CR_y and R_y gates in half as compared to the $k=1$ RUS neuron, remove the CY and R_z gates, and do not require repeating the circuit execution due to failure. Our gate complexity therefore reduces to $(10n-3)u$ total gate operations, or approximately $O(nu)$. This depth breaks down to $(8n-4)u$ single qubit gates and $(2n+1)u$ $CNOT$ gates, both significantly less than the gate complexity of the RUS neuron. Our neuron complexity is further improved by not requiring non-unitary reset and measurement operations or any classical conditioning, which the RUS

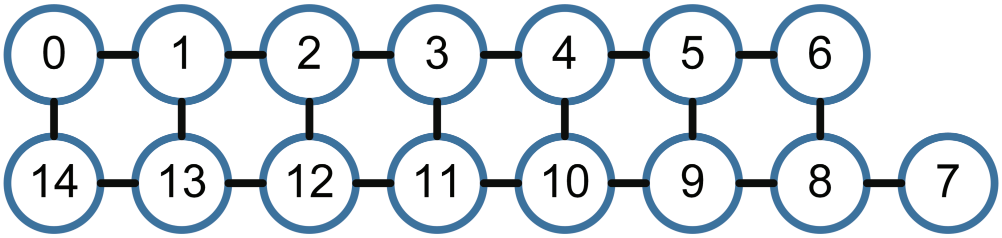

Figure S1. Melbourne hardware qubit architecture. Hardware architecture of the IBMQ *ibmq_16_melbourne* system, available from IBMQ.[2,4]

Table S1. Measured noise characteristics of IBMQ hardware. Noise and error modeling characteristics of IBMQ hardware used to create their various noise models.[4–11]

Device	Number of Qubits	Processor	Average T_1 Time (μs)	Average T_2 Time (μs)	Avg Readout Error Rate (%)	Avg Single Qubit SX Error Rate (%)	Avg Two Qubit CNOT Error Rate (%)	Quantum Volume
ibmq_16_melbourne	15	Canary	55.60	56.15	6.89	0.125	3.05	8
ibmqx2*	5	Canary	59.30	36.05	4.58	0.099	1.75	8
ibmq_athens	5	Falcon	74.08	91.22	2.02	0.045	1.21	32
ibmq_santiago	5	Falcon	121.58	101.01	4.36	0.024	0.74	32
ibmq_lima	5	Falcon	79.79	85.86	2.60	0.034	0.97	8
ibmq_quito	5	Falcon	81.83	80.41	2.92	0.054	1.21	16
ibmq_belem	5	Falcon	75.62	100.24	2.56	0.026	1.19	16
ibmq_armonk	1	Canary	138.19	222.74	2.60	0.019	N/A	1

*Also known as ibmq_yorktown

neuron requires with each failure of the RUS construction. Additionally, Cao et al. propose performing many iterations of the same neuron in order to take a majority vote of the measurement results and reassign the neuron value according to the most frequently measured result. Since our design proceeds with all necessary updates without stopping to measure the output of each neuron mid-circuit execution, our design also requires significantly less repetition and measurement.

IBMQ Melbourne Hardware Architecture

The architecture of the *ibmq_16_melbourne*[2,4] system in IBMQ shown in Fig. S1 is a grid architecture, meaning that no single qubit is directly physically connected to any more than three other qubits. To route quantum gate operations between any qubits which are not directly connected, intermediary operations are used by the IBMQ Transpiler to route the operation through the connecting qubits from the control qubit to the target qubit. This routing often consists of many quantum SWAP gates, each composed of three CNOT gates, which can be used to swap the quantum states of any two connected qubits. For example, a CNOT gate implemented with qubit 0 as the control and qubit 2 as the target using the *ibmq_16_melbourne* device shown in Fig. S1 would need to first route through qubit 1, causing the need for additional gates and thus a higher error rate than a comparable CNOT gate implemented on neighboring qubits.

IBMQ Noise Model Parameters

We utilize noise models created by IBMQ to study the impact of quantum hardware noise on our neuron and QHAM. IBMQ lists average thermal relaxation (T_1) and dephasing (T_2) times[12] for each qubit, average single-qubit gate error rates for each qubit (measured by the average error rate of the Sqrt-X “SX” gate), average two-qubit gate error rates for each two qubit control-target pattern (measured by the average error rate of the CNOT gate), and average readout errors of each qubit for all of its hardware devices.[4–11] Using these characteristics, noise models are created which are used to approximate errors resulting from operating circuits in hardware. A summary of parameters used in these noise models is shown in Table S1. Overall, the

Table S2. Noisy QHAM results using hardware and simulated noise models. Comparison of results from hardware and simulation with various noise models[4–11] for the $n = 4$ associative memory configuration tested in Fig. 6. In all cases, the only qubit which is chosen to be updated is Qubit 3.

Device	Qubit 1	Qubit 2	Qubit 3	Qubit 4	Avg Accuracy (%)
Target Result	0.0	1.0	1.0	0.0	100.0
Noiseless Simulation	0.0	1.0	1.0	0.0	100.0
ibmq_16_melbourne Noise Model	0.011	0.94	0.94	0.065	95.1
ibmqx2* Noise Model	0.024	0.95	0.93	0.023	95.8
ibmq_athens Noise Model	0.0089	0.97	0.96	0.0052	97.9
ibmq_santiago Noise Model	0.0095	0.98	0.97	0.068	96.8
ibmq_lima Noise Model	0.0089	0.98	0.97	0.014	98.2
ibmq_quito Noise Model	0.024	0.97	0.95	0.025	96.8
ibmq_belem Noise Model	0.037	0.97	0.96	0.013	97.0
ibmq_16_melbourne Hardware	0.10	0.70	0.67	0.049	80.5

*Also known as ibmq_yorktown

combined error rates can be used to calculate a metric called quantum volume[13] which quantifies the largest random quantum circuit of equal width and depth that each system can successfully implement. Naturally, this metric tends to be larger for systems with low gate and readout error rates and more advanced backend processing. However, this metric does not take physical hardware connectivity into account, leading to significant differences in noise model simulation and true hardware results, as shown in Figs. 8 and 9. and Table S2 For much of our QHAM testing we choose to use *ibmq_16_melbourne* because of its large number of physical qubits, but for smaller circuits it can be wise to use systems such as *ibmq_athens* or *ibmq_santiago* because of their larger quantum volume.

Noisy QHAM Simulation

We perform the $n = 4$, single update test of Figs. 6 and 4a using each of the available noise models to benchmark the performance of the update step with respect to noise, as shown in Table S2. The average accuracy shown in the table is calculated as the average bitwise accuracy of the four qubits compared to their target value of $|0110\rangle$. It is clear that the final accuracy of the associative memory system depends greatly on the average error rates of the hardware. For example, *ibmq_16_melbourne* and *ibmqx2* are the systems with the lowest T_1 and T_2 times, highest readout error rates, and highest single-qubit and two-qubit gate error rates, and as expected, they show the worst overall accuracy for the results shown in Table S2. However, it is interesting that *ibmq_lima* shows the highest average accuracy in this test, even though it has a lower quantum volume[13] than *ibmq_athens*, *ibmq_santiago*, *ibmq_quito* and *ibmq_belem*.

Since the qubit connectivity of the hardware system on which the QHAM is executed is crucial for performance, and as Fig. 6 shows, the accuracy of the QHAM in hardware can be worse than in simulation. For example, the *ibmq_16_melbourne* hardware test shown in Fig. 6 clearly performs worse than simulations with the corresponding noise model. This is explained by the hardware execution of the circuit requiring additional operations and qubits than simulation. For the system we are modeling with a QHAM of length $n = 4$ with a single update, we require 5 qubits which must all be connected to complete the update. However, there is no qubit in the *ibmq_16_melbourne* hardware which is directly connected to four other qubits, so performing the desired update requires routing the control signals through multiple qubits to reach their targets. This process

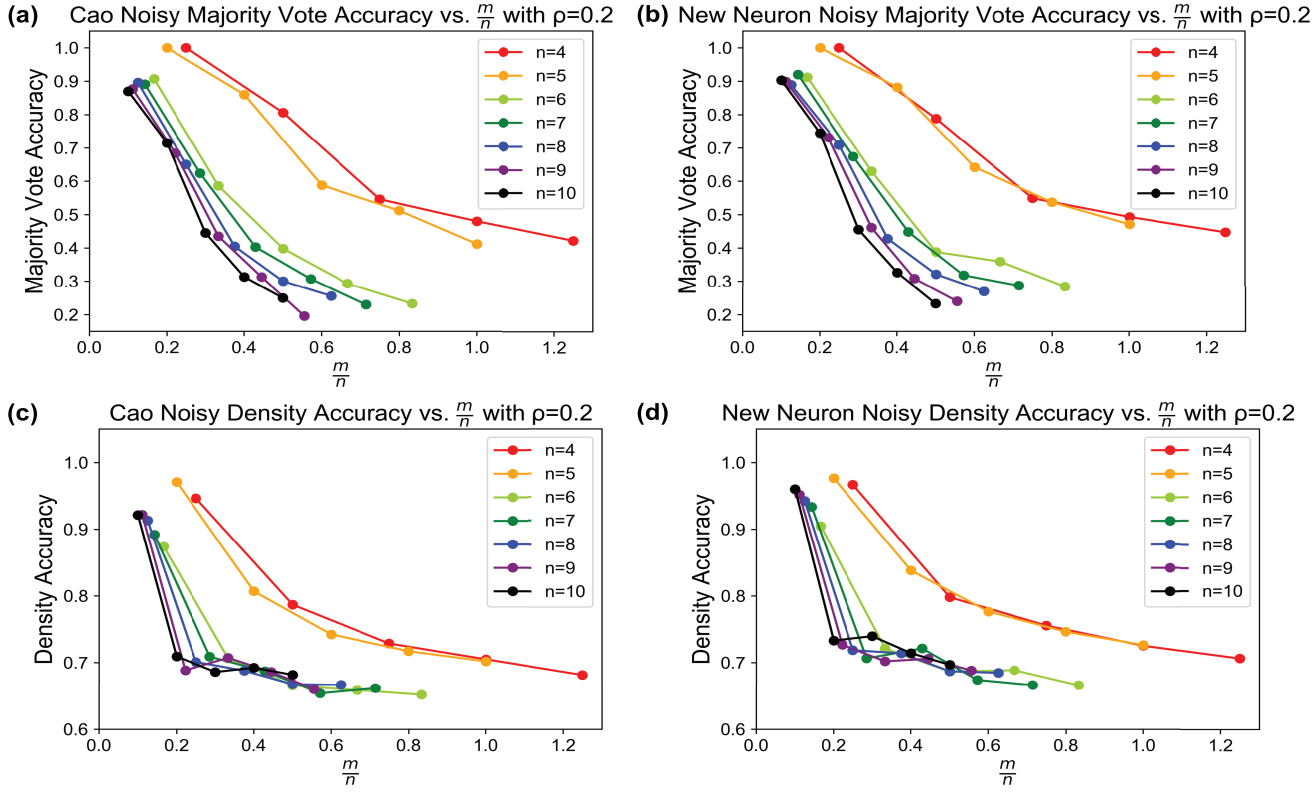

Figure S2. Effective memory capacity simulation of both neuron systems. Simulations of the effective memory capacity of associative memories built from the Cao et al.[1] neuron and our neuron using the *ibmq_quito* noise model following the same process as Figs. 7 and 8.

requires several SWAP gates, and errors from each operation tend to compound and increase the error rate. Thus, the hardware performance of our QHAM is highly dependent on the overall connectivity of the qubits.

Comparing the Effective Memory Capacity of Both Quantum Neuron Systems

We benchmark our QHAM against an associative memory created using the quantum neuron by Cao et al.[1] in order to understand how the addition of simulated noise (using the *ibmq_quito* noise model) affects both systems, as shown in Fig. S2. The same process of testing effective memory capacity and tuning with the u parameter as in Figs. 7 and 8 is implemented for $\rho = 0.2$, $n \in [4,5,\cdots,10]$ and $m \in [1,2,3,4,5]$. Both networks have similar effective memory capacities which, in the majority vote accuracy metric, closely mirror the expected memory capacity from classical associative memories as described by equation 9. As we expected from the results of Fig. 3, our network shows a minor improvement in both majority vote accuracy and density accuracy in nearly all cases of around 5 %. Even though our neuron response in Fig. 3 is flatter than the Cao et al. neuron response, our network still sees this minor improvement due to its resiliency to noise caused by a lower gate complexity.

References

1. Cao, Y., Guerreschi, G. G. & Aspuru-Guzik, A. Quantum neuron: an elementary building block for machine learning on quantum computers (2017). 1711.11240.
2. IBM Quantum. https://quantum-computing.ibm.com/ (2021).
3. ibmq_qasm_simulator v0.1.547, IBM Quantum team. Retrieved from: https://quantum-computing.ibm.com/ (2021).
4. ibmq_16_melbourne v2.3.8, IBM Quantum team. Retrieved from: https://quantum-computing.ibm.com/ (2021).
5. ibmq_5_yorktown (ibmqx2) v2.2.6, IBM Quantum team. Retrieved from: https://quantum-computing.ibm.com/ (2021).
6. ibmq_lima v1.0.2, IBM Quantum team. Retrieved from: https://quantum-computing.ibm.com/ (2021).
7. ibmq_quito v1.0.4, IBM Quantum team. Retrieved from: https://quantum-computing.ibm.com/ (2021).
8. ibmq_belem 1.0.0, IBM Quantum team. Retrieved from: https://quantum-computing.ibm.com/ (2021).
9. ibmq_athens v1.3.10, IBM Quantum team. Retrieved from: https://quantum-computing.ibm.com/ (2021).
10. ibmq_santiago v1.3.10, IBM Quantum team. Retrieved from: https://quantum-computing.ibm.com/ (2021).
11. ibmq_armonk v2.4.0, IBM Quantum team. Retrieved from: https://quantum-computing.ibm.com/ (2021).
12. Kofman, A. G. & Kurizki, G. Unified theory of dynamically suppressed qubit decoherence in thermal baths. *Phys. Rev. Lett.* **93**, 130406, DOI: 10.1103/PhysRevLett.93.130406 (2004).
13. Cross, A. W., Bishop, L. S., Sheldon, S., Nation, P. D. & Gambetta, J. M. Validating quantum computers using randomized model circuits. *Phys. Rev. A* **100**, DOI: 10.1103/physreva.100.032328 (2019).
